# Phylogenetic Reconstruction Shows Independent Evolutionary Origins of Mitochondrial Transcription Factors from an Ancient Family of RNA Methyltransferase Proteins

**DOI:** 10.1007/s00239-018-9842-z

**Published:** 2018-04-25

**Authors:** AJ Harris, Aaron David Goldman

**Affiliations:** 1 Department of Biology, Oberlin College and Conservatory, K123 Science Center, 119 Woodland Street, Oberlin, OH 44074 USA; 2 Blue Marble Space Institute of Science, Seattle, WA 98154 USA

**Keywords:** Dimethyladenosine transferase, Mitochondrial transcription factor, LUCA, Neofunctionalization, rRNA adenine *N*(6)-methyltransferase, Tree of life

## Abstract

Here, we generate a robust phylogenetic framework for the rRNA adenine *N*(6)-methyltransferase (RAMTase) protein family that shows a more ancient and complex evolutionary history within the family than previously reported. RAMTases occur universally by descent across the three domains of life, and typical orthologs within the family perform methylation of the small subunits of ribosomal RNA (rRNA). However, within the RAMTase family, two different groups of mitochondrial transcription factors, mtTFB1 and mtTFB2, have evolved in eukaryotes through neofunctionalization. Previous phylogenetic analyses have suggested that mtTFB1 and mtTFB2 comprise sister clades that arose via gene duplication, which occurred sometime following the endosymbiosis event that produced the mitochondrion. Through dense and taxonomically broad sampling of RAMTase family members especially within bacteria, we found that these eukaryotic mitochondrial transcription factors, mtTFB1 and mtTFB2, have independent origins in phylogenetically distant clades such that their divergence most likely predates the last universal common ancestor of life. The clade of mtTFB2s comprises orthologs in Opisthokonts and the clade of mtTFB1s includes orthologs in Amoebozoa and Metazoa. Thus, we clearly demonstrate that the neofunctionalization producing the transcription factor function evolved twice independently within the RAMTase family. These results are consistent with and help to elucidate outcomes from prior experimental studies, which found that some members of mtTFB1 still perform the ancestral rRNA methylation function, and the results have broader implications for understanding the evolution of new protein functions. Our phylogenetic reconstruction is also in agreement with prior studies showing two independent origins of plastid RAMTases in Viridiplantae and other photosynthetic autotrophs. We believe that this updated phylogeny of RAMTases should provide a robust evolutionary framework for ongoing studies to identify and characterize the functions of these proteins within diverse organisms.

**Electronic supplementary material:**

The online version of this article (10.1007/s00239-018-9842-z) contains supplementary material, which is available to authorized users

The enzymatic rRNA adenine *N*(6)-methyltransferase protein (RAMTase) family occurs in all three domains of life and is responsible for the methylation of two adjacent adenosine molecules in the highly conserved 3′ terminal hairpin loop of ribosomal small subunits (rSSU; O’Farrell et al. 2006, 2008). The family is also known as DIM for its dimethyltransferase activity as well as KsgA for conferring resistance to kasugamycin in bacteria, in which the dimethylation activity was originally characterized (Formenoy et al. 1994; Helser et al. 1971; Lafontaine et al. 1994). RAMTase is dependent on S-adenosylmethionine, or SAM, from which it obtains four methyl groups to split between its two adenosine targets (McCulloch et al. 2002). SAM is the most common source of methyl groups in living systems and can deliver methyl groups to proteins with conserved SAM domains (Cheng and Roberts 2001; Lu 2000). RAMTases methylate an intermediate of the rSSU and are necessary for its maturation (Connolly et al. 2008; Desai and Rife 2006; Lafontaine et al. 1998; O’Farrell et al. 2006). They may also help to optimize mature rSSUs for protein synthesis (Connolly et al. 2008; Gregory et al. 2011). In addition to the presence of RAMTases within the three domains of life, proteins of this family were likely also encoded in the genomes of the endosymbiotic progenitors of plastids and mitochondria (Lisowsky and Michaelis 1988; Patron et al. 2005; Rife 2009; Shutt and Gray 2006). In extant eukaryotes, plastid and mitochondrial RAMTase genes have been transferred to the nucleus, but their protein products localize to the respective organelles where they retain their function of methylating organellar rSSUs (Lisowsky and Michaelis 1988; Park et al. 2009; Patron et al. 2005).

Among eukaryotic lineages, the Metazoa (i.e., animals) are unique in having two RAMTase orthologs that localize to the mitochondria (Falkenberg et al. 2002; Manna and Harman 2014; McCulloch et al. 2002). Of these orthologs, one, mtTFB2, has undergone neofunctionalization and plays a role in transcription of the mitochondrial genome. The other, mtTFB1, has retained its methylation function and is required for biosynthesis of the mitochondrial rSSU and translation (Matsushima et al. 2005; Metodiev et al. 2009; Shutt et al. 2010, 2011). The presence of these two orthologs are particularly well documented in model organisms, especially humans, *Drosophila melanogaster* Meigen (fruit flies), and *Caenorhabditis elegans* Maupas (nematodes) (Falkenberg et al. 2002; Matsushima et al. 2005). Outside of Metazoa, mtTFB proteins are documented in single copy, such as in sampled lineages of Fungi, Aveolata, and “Excavata”^1^ (Richter et al. 2010; Shutt and Gray 2006), where it appears to function primarily in transcription, not methylation, (Shadel and Clayton 1995), and Amoebozoa, where it appears to be essential for both methylation and transcription (Manna et al. 2013). No mtTFB orthologs of RAMTases have been detected in Viridiplantae (green plants) or Rhodophyta (red algae).

Prior phylogenetic analyses of the RAMTase family depict several complex evolutionary events, especially related to the presence of the family in eukaryotic lineages (Cotney and Shadel 2006; Park et al. 2009; Shutt and Gray 2006). Notably, all mtTFBs with neofunctionalized roles solely or primarily in transcription comprise a clade in which the mtTFB2s of Metazoa and the mtTFBs of other lineages (hereafter mtTFB2s) are mutually monophyletic (Cotney and Shadel 2006; Park et al. 2009; Shadel and Clayton 1995; Shutt and Gray 2006). Moreover, these phylogenies show that the mtTFB-mtTFB2 clade is sister to mtTFB1s, suggesting a duplication prior to divergence of a eukaryotic lineage comprised at least of Fungi, Metazoa, Aveolata, and “Excavata” (Shutt and Gray 2006; see tree in; Burki 2014). The mtTFB1 clade includes the duo-functional amoebozoan mtTFB (hereafter, mtTFB1) based on phylogeny and common domain architecture (Manna et al. 2013; Shutt and Gray 2006). Additionally, previous phylogenetic analyses found that the RAMTases that localize to plastids entered the eukaryotic lineage at least twice: once within Viridiplantae and once within all other sampled plastid-bearing lineages, such as Rhodophyta, some “Excavata,” and Alveolata (Park et al. 2009). Thus, the origins of the RAMTases that localize to plastids appear inconsistent with both eukaryotic and plastid evolution, in which Viridiplantae and Rhodophyta are sister phyla that obtained their plastids through a single endosymbiosis event involving a bacterium, while the other lineages trace their plastids to secondary or tertiary endosymbiosis events involving a photosynthetic eukaryote (McFadden and van Dooren 2004). Overall, the origins of the RAMTase protein family have not yet been well-resolved within the three domains of life, because prior phylogenetic studies were constrained by limited sampling of bacteria, which may harbor considerable ancient protein diversity due to their antiquity and vast biodiversity (Hug et al. 2016; Nemergut et al. 2011) and which are known to play important roles in lateral evolutionary mechanisms.

Our phylogeny of the RAMTase family comprises 730 unique sequences representing 651 bacterial accessions, 47 eukaryotic accessions, and 31 archaeal accessions (Supplementary File 1). Our phylogenetic results (Fig. 1, Supplementary Files 2, 3) show a deep gene duplication within the RAMTase family predating the last universal common ancestor, or LUCA, of the three domains of life. Thus, our results suggest that RAMTases are one of the few universal paralogs preserved within the tree of life and, consequently, confirm that rRNA methylation mediated by SAM was probably important within the LUCA (Goldman et al. 2010, 2012; Weiss et al. 2016). Of the paralogs, Lineage 1 is present within all three domains of life by descent while Lineage 2 was lost in all but Bacteria, from which it later reentered eukaryotic lineages by lateral mechanisms. Unlike previously published phylogenies of this family, our phylogeny shows strongly supported independent origins within bacteria of the mtTFB orthologs, with all mtTFB2s that act as transcription factors resolved within Lineage 1 and the methylating mtTFB1s along with the bifunctional Amoebozoan mtTFB1 resolved within Lineage 2. Both lineages of mtTFBs are most closely related to proteobacterial orthologs. Therefore, Lineages 1 and 2 were probably both present in the ancestral Proteobacterium that was the progenitor of the mitochondrial endosymbiont (Kurland and Andersson 2000) were laterally transferred into the eukaryotic domain via the mitochondrial endosymbiosis event. Additionally, our phylogeny is consistent with a prior study (Park et al. 2009) showing a complex relationship among RAMTases originating from plastids in Virdiplanteae and other photosynthetic eukaryotes, here represented by photosynthetic Chromerida and vestigial-plastid-bearing Apicomplexa (Sato 2011) of the Alveolata. The plastid RAMTases of both Virdiplanteae and other photosynthetic eukaryotes evolved within Lineage 2 but are only distantly related to one another (Fig. 1; Supplementary File 2, 3). Specifically, Viridiplantae plastid RAMTases are sister to a clade of *Chlamydia* Jones et al. 1945 emend. Everett et al. 1999, which has been implicated as an essential mediator of primary endosymbiosis (Ball et al. 2013), but see (Domman et al. 2015), and plastid RAMTases of Alveolata are sister to *Salinibacter ruber* Antón, an extreme halophyte (Oren 2013) that may have shared genes laterally with the cyanobacterial progenitor of plastids prior to the primary photobiotic endosymbiosis event (Gross et al. 2008).

**Fig. 1:**
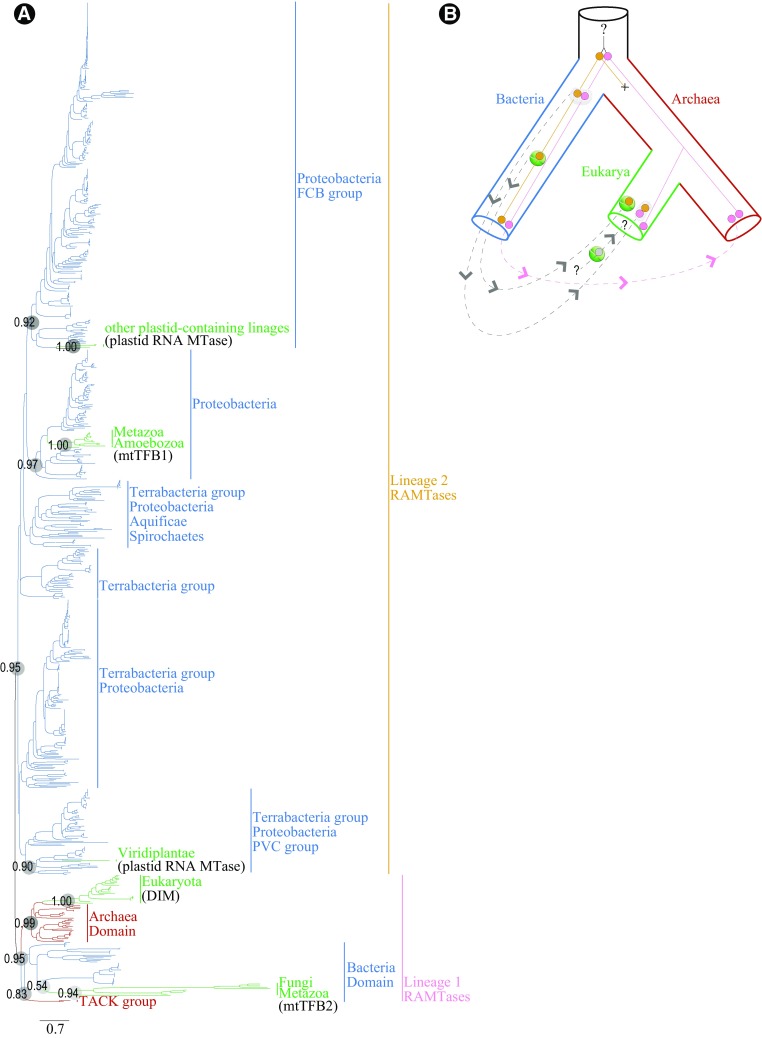
Phylogeny of the RAMTase gene family across the three domains of life. **a** Maximum likelihood tree reconstructed from 722 reviewed accessions ascribed to the rRNA adenine *N*(6)-methyltransferase family in Uniprot (http://www.uniprot.org/; Accessed 20 March 2018) and supplemented with seven sequences of phylum Aveolata from Genbank (https://www.ncbi.nlm.nih.gov/genbank/), selected by a standard protein BLAST via the web portal using a plastid-localizing RAMTase from *Arabidopsis thaliana* (Uniprot: O65090) as a query and limiting results to Aveolata and Euglenozoa. We selected the top hits with 75% or better coverage and having e-values of 0.001 or better. We performed the analysis using FastTree v.2.1.5 (Price et al. 2010) implemented in Geneious (Kearse et al. 2012) and show local bootstrap support values that constrain the topological locations of relevant clades in gray circles on branches. The ML tree shows congruent results with an analysis using Neighbor Joining (Supplementary File 3). Clades of Bacteria, Archaea, and Eukarya are labeled to the left of color coded branches in blue, red, and green, respectively, and functions of proteins in each clade given in black for Eukarya. Lineages 1 in pink and 2 in orange represent the fates of ancestral paralogs of RAMTase. **b** Domain-level coalescent hypothesis for the RAMTase family. Domain (species) tree is shown as a large 3-dimensional tree and thin lines within represent hypothesized protein histories. Colors used to represent domains and protein lineages are the same as in **a**. “X” shows lineage loss, dashed lines represent lateral transfers of orthologs, and orthologs transferred within mitochondria and plastids are shown within representations of the organelles. Unknowns are presented by question marks; especially parts of the plastid history and events prior to the duplication of Lineages 1 and 2

This new, robustly supported phylogenetic framework has implications for better understanding the evolution of new functions in the RAMTase protein family, especially the mtTFB orthologs, and suggests areas in which additional experimental and bioinformatics research is needed. Here we show vast phylogenetic and temporal distance between the lineages containing mtTFB1 and mtTBF2, which were duplicated prior to the divergence of the LUCA roughly 3.8–2.9 billion years ago (Caetano-Anollés et al. 2014; Wacey et al. 2011). The fact that similar transcriptional regulator functions emerged independently from the ancestral RNA methyltransferase function has important implications for understanding neofunctionalization and functional evolvability in protein families. This result suggests that the ancestral rRNA methyltransferases may have been especially amenable to neofunctionalization as transcriptional regulators perhaps because of their particular mode of nucleic acid binding during the process of methylation. An alternative interpretation of the phylogeny presented here is that the ancestral protein was capable of both transcription and methylation and that subsequent subfunctionalization has produced single function proteins (Des Marais and Rausher 2008; Hughes 1994; Lynch and Conery 2000), the majority of which have retained the RNA methylations function. This second scenario is consistent with possible biases in the genome of the LUCA and its predecessors towards genes with multifunctionality, at least for some cellular processes (Ranea et al. 2006). The ability of exclusively methylating mtTFB1s to perform transcription in vitro (Falkenberg et al. 2002) and the bifunctionality of the Amoebozoan mtTFB1 can both be interpreted as supporting either of these scenarios. Future experimental characterization of orthologs across the RAMTase tree will help to resolve the evolutionary history of protein functions within the RAMTase family.

Our results also highlight many remaining uncertainties regarding the origins of RAMTases in plastid-bearing eukaryotic lineages (Fig. 1b). Presently, it is difficult to develop a hypothesis for the origins of plastid-associating RAMTases. The evolutionary history of plastid organelles among eukaryotic lineages is considerably less certain than that of mitochondria and is also possibly more complex due to multiple endosymbiosis events (Archibald 2009; Ball et al. 2013; Domman et al. 2015; Gross et al. 2008). Moreover, the evolutionary history of RAMTases in plastid-bearing lineages is also compounded by at least one duplication of Lineage 1 detected within *Arabidapsis thaliana* (L.) Heynh.; DIM1B, which localizes to the mitochondria, performs methylation, and, thus, behaves like an mtTFB1 ortholog (Richter et al. 2010) (see also Supplementary File 2, Uniprot accessions 022268 and Q9FKO2). A better understanding of RAMTases in plastid-bearing lineages can most likely be achieved through an improved evolutionary framework for plastid evolution as well as additional identification and characterization of RAMTases in plastid-bearing model and non-model organisms.

## Electronic supplementary material

Below is the link to the electronic supplementary material.

Supplementary File 1 Alignment of RAMTases used to generate phylogenetic trees. File includes accession details for Uniprot and Genbank as well as taxonomic identities. In Nexus format. (NEX 588 KB)

Supplementary File 2 Maximum likelihood phylogenetic tree of RAMTases described and shown in Figure 1. File includes accession details for Uniprot and Genbank as well as taxonomic identities. In Nexus format; contains all support values. (NEX 98 KB)

Supplementary File 3 Neighbor joining (NJ) tree of RAMTases showing same groups as in maximum likelihood tree (Fig. 1, Supplementary File 2) but with lower support and unstable backbone topology. NJ tree generated using proprietary algorithm in Geneious with JC model and 1000 bootstrap replicates to estimate support for branches. File includes accession details for Uniprot and Genbank as well as taxonomic identities. In Nexus format; contains all support values. (NEX 162 KB)

## Footnotes

1 Throughout, we use National Center for Biotechnology Information (NCBI) taxonomy except for the "Excavata" group, which is split into smaller Phyla within the eukaryotic domain but treated as a single entity in prior studies of this protein family. We place "Excavata" in quotation marks to acknowledge that it is not recognized by NCBI and may not be monophyletic (Burki 2014; Hampl et al. 2005; Simpson et al. 2006).
